# Ethical perspectives on GPS tracking for people with dementia: insights from an online citizens’ jury

**DOI:** 10.1186/s12910-026-01423-5

**Published:** 2026-03-03

**Authors:** Isabell Strobl, Ruben Andreas Sakowsky, Mark Schweda, Silke Schicktanz

**Affiliations:** 1 Department of Medical Ethics and History of Medicine, University Medical Center Göttingen, Humboldtallee 36, Göttingen, 37073 Germany; 2 Faculty of Health Sciences Brandenburg, University of Potsdam, Am Mühlenberg 9, Haus 62, Potsdam, 14476 Germany; 3 Department of Health Services Research, Division for Ethics in Medicine, School of Medicine and Health Sciences, Carl von Ossietzky University of Oldenburg, Ammerländer Heerstr. 114-118, Oldenburg, 26129 Germany

**Keywords:** Dementia, GPS monitoring, Assistive technology, Medical ethics, Digital health ethics, Participatory research, Deliberative methods, Citizens’ jury, Autonomy, Privacy

## Abstract

**Background:**

The growing number of older adults with dementia is increasingly being met with technological interventions. Among them are GPS-tracking devices that assist people with dementia (PwD) with orientation. The extent to which this technology should be utilized, and how to balance safety, mobility, and privacy has been the subject of extensive scholarly debate.

**Methods:**

To evaluate the range of normative attitudes of lay people towards the issue, we conducted an online citizens’ jury (CJ) with 17 German citizens aged 18 to 67. To examine the relationship between individual perspectives and collective attitudes, we further conducted qualitative interviews with participants. As part of the CJ process, participants collaboratively authored a comprehensive policy proposal containing nuanced views on ethical and technical aspects.

**Results:**

Despite differing perspectives, particularly on the meaning of autonomy, participants reached broad consensus on issues such as informed consent, data protection, and device customization, and were able to agree to disagree on more contentious points.

**Conclusions:**

Our analysis demonstrates the capacity of lay participants to engage in ethical reflection, achieve consensus, and navigate disagreement. We further reflect on methodological considerations and examine the contribution lay deliberation can provide to contemporary bioethical debates. The online CJ format enabled in-depth, structured deliberation among diverse lay participants on a complex bioethical topic. The use of video conferencing software allowed for wide geographic participation. Combining the CJ with qualitative interviews and field notes provided rich insight into both collective and individual normative attitudes.

**Supplementary Information:**

The online version contains supplementary material available at 10.1186/s12910-026-01423-5.

## Background

By 2022, 1,8 million people in Germany were diagnosed with dementia [3]. As the disease progresses, orientation problems may occur. Between 15—60% of people with dementia (PwD) will exhibit ‘wandering’ behaviour, in which PwD walk about with no apparent goal and may get lost and incur serious harm [5, 17, 18, 21]. Various methods of managing orientation issues and wandering have been employed, including physical barriers, physical restraints, and drugs [21], as well as architectural design built to influence PwD’s behaviour [1]. The increased use of technological devices in the care of older adults means that such devices can also be used to assist PwD when dealing with orientation problems. In particular, the use of GPS-assisted tracking systems is discussed as a possible orientation aid for PwD and as a tool to provide caregivers with information on the whereabouts of PwD [6, 14].

The use of this technology raises ethical issues. The concern for the safety of PwD on the one hand and respect for their autonomy and privacy on the other, represents the major conflict that we will address in this contribution. While the acceptability of the technology among PwD and caregivers is generally high [6], the bioethical debate has identified a number of potential ethical issues, mainly centering around concerns about the reliability and usability of the systems; greater liberty for PwD when compared to more restrictive measures; safety benefits; reassurance and a sense of safety for PwD, carers and relatives; potential violations of PwD’s privacy, autonomy, and dignity; and issues of consent [6, 14, 17–19]. A systematic review of the normative literature on the issue [11] revealed two main themes emerging from the expert discourse: First, the dual effect of tracking devices, potentially leading to both an increase and a decrease in the ability of PwD to exercise their autonomy, and second, a focus on the trade-off between autonomy and safety. The review further identified the lack of focus on the design of tracking devices as a major gap in the literature in need of further attention.

Explorative qualitative research by [27] suggests that caretakers may prioritize safety concerns over autonomy, suggesting that their attitudes differ significantly from those of some experts. Given the anticipated increase in dementia patients in the coming years [3], it is important to investigate the normative attitudes of members of the general public towards this potentially contentious issue. As new policies will be needed to navigate complex trade-offs between safety, autonomy, and privacy, it is paramount to investigate not only the normative positions of members of the public, but also elicit the reasons for the positions they hold and the trade-offs they are willing to make when normative goods collide. Deliberative methods that focus on participants exchanging normative arguments and learning about their peers’ points of view, are particularly suited to investigate this complex interplay [7].

To elicit the attitudes of laypersons towards the use of GPS systems for PwD and related ethical issues, we conducted an online Citizens’ Jury (CJ) with 17 members of the German public. While CJs are traditionally held face-to-face, we developed a novel online-only approach for our deliberative setting, allowing us to significantly broaden the pool of prospective participants. We present and analyse the collective normative position of participants and compare them to individual attitudes elicited via semi-structured qualitative interviews with participants after the conclusion of the CJ. Combining deliberative group discussion methods with in-depth qualitative interviews and non-participatory observation allowed us to contrast individual attitudes with the consensus produced by the deliberative event, gaining insights into the relative strength of ethical concerns and participants’ willingness to compromise and trade-off. Our methodology also sheds light on the impact of deliberation on the process of individual attitude formation and reasons for changes of mind as well as on the feasibility of conducting deliberative events via Zoom.

## Methods

### Citizens’ Jury

Following a pilot study in which feasibility, structure, and methods of our online approach were tested with local students on the topic of pandemic-apps (Bürgerforum forschungsorientierte Pandemie-Apps, [4]), we employed the approach to discuss the attitudes of German citizens towards the use of GPS systems for PwD. The aim of the CJ was to facilitate deliberation among citizens in order to explore normative attitudes and reasoning processes. We use the term ‘citizens’ jury’ to refer to the deliberative format employed, rather than to denote a claim regarding community reflectiveness in jury composition. The online-only method was chosen to broaden the geographic reach of our approach as well as to approach individuals who would otherwise have been incapable of attending in-person events, such as people with work or care obligations. A call for applications was disseminated country-wide via social media advertisements (Facebook, Instagram), and locally via flyers. The material advertised the topic of the CJ and contained a QR code link, directing potential participants to an application page containing questions about their demographics (age, sex, education, profession) and whether they had access to a stable internet connection, a microphone, camera, and a sufficiently large screen for the duration of the CJ. We received 74 applications. Three applications were excluded during initial screening (one due to incompleteness and two due to not meeting the technical equipment requirements), leaving 71 eligible applicants. The remaining candidates were allocated to lottery pots according to their age group, gender, and educational background. Random draws alternated between pots to ensure a balance between the aforementioned criteria (Table 2). 20 participants were subsequently invited, of whom three withdrew prior to participation. These individuals did not differ markedly from the final sample based on the available demographic information.

Participants received an information package in the form of a brochure providing general information on the topic of the CJ. The information material included a short introduction of the team and information on the selection process, the structure of CJs, the prevalence of dementia and explicitly on GPS tracking and wandering. In addition, the team's research interest was made transparent, namely to research attitudes towards the use of GPS systems as well as mechanisms of opinion formation in deliberation. General information on the schedule and a loose code of conduct were provided. For the online meetings, participants were asked to identify and discuss ethical issues connected to the use of GPS systems for PwD, and to produce a set of policy recommendations [26]. The CJ comprised five meetings with a total duration of 16 h and one voluntary meeting of a smaller group of participants to finalise the policy recommendations document in accordance with the whole group’s wishes. The meetings were held in May and June 2022 (05/04/2022–06/09/2022). Initially, four sessions were scheduled, but an additional appointment was organized at the request of participants. Up until the 3rd meeting, all participants were present; on the fourth meeting, one participant dropped out. The last additional appointment, scheduled at short notice, was attended by 10 of the initial 17 participants. The discussion was facilitated by two members of the research team (SiSchi and MS, RS for an additional session) to encourage an open and fair culture of debate and help with time management. Participants received an honorarium of €100 for their time.

**Fig. 1:**
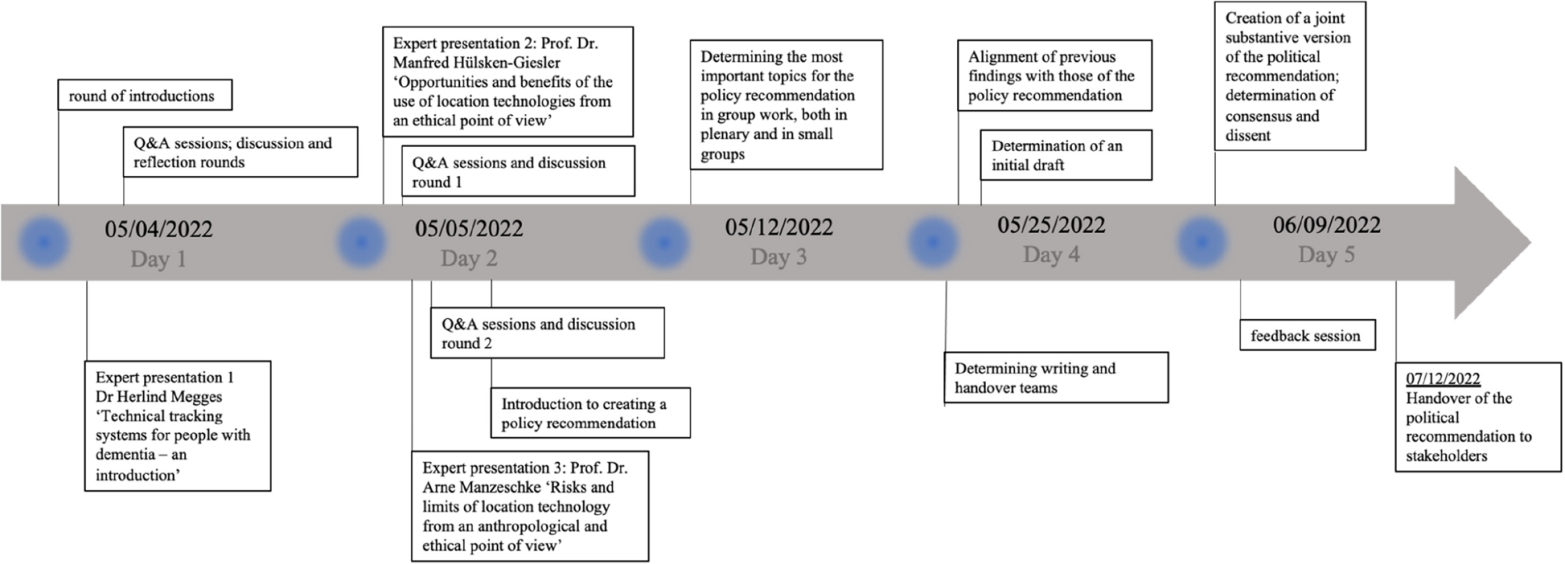
Structure of the CJ

The CJ was held entirely online, utilizing the video conference software *Zoom* and collaborative whiteboard software *Miro*. During the first two sessions, participants were provided with three external expert lectures with subsequent Q&A sessions (Table 1, Fig. 1). The three experts were selected for their expertise in the fields of gerontology, care science, and health ethics. The lectures focused on, respectively, (1) general information on dementia and GPS tracking devices including application examples, (2) potential benefits of utilising GPS tracking with a focus on enabling safe mobility, enhancing self-determination, and decreasing the burden for caregivers, and (3) potential ethical issues with the technology, especially given that tracking systems are assistant systems for caregivers rather than PwD. Beginning on day 2, participants discussed their positions in facilitated and unfacilitated discussion rounds both among the whole group and in smaller groups. Over the course of the process, participants anonymously noted their viewpoints and questions on the digital white board software, creating a repository of positions readily available for the whole group. This helped facilitate the process of identifying and discussing important open questions, identifying normative positions of group members, as well as finding consensus and points of contention. This process was designed to be iterative and featured multiple rounds of discussion in various group settings with various compositions. In response to the participants' requests, an additional information package on technical and legal aspects of using GPS tracking systems was prepared and handed out after the 2nd day. Participants were asked to formulate a policy recommendation document describing their views and presenting their recommendations with respect to the use of GPS systems for PwD. Participants produced an extensive and nuanced report. The participants outlined the structure and content of the document and subsequently elected a writing team of 4 participants to finalize the text with regarding to language, editing issues and to avoid misunderstandings. CJ organizers provided layout and proofreading support. The final report consists of a 15-page document which was published online (Bürgerforum forschungsorientierte Pandemie-Apps, [4]). On July 12th, 2022, it was presented to a group of representatives from stakeholder organisations and researchers involved in the technological development of GPS systems: The National Academy of Science and Engineering, The German Nursing Association, the German National Association of Senior Citizens’ Organisations, and the German Alzheimer Association.

**Table 1:** Expert speaker input

*Expert name*	*Occupation/Expertise*	*Input provided*
Dr. Herlind Megges	Consultant at the Federal Ministry for Family Affairs, Senior Citizens, Women and Youth	Introduction to dementia: prevalence, different forms of dementia, care demands, disorientation issues (“wandering”) and their progression, consent and the ability to consent, explanation of various GPS devices and functions, including advantages and disadvantages
Prof. Dr. Manfred Hülsken-Giesler	Professor at the University of Osnabrück, Department of Nursing Science	Expert was tasked with taking a more favourable stance towards the use of GPS systems. Opportunities and benefits of using location technology from an ethical perspective, relationship between dementia and movement, challenges of increased mobility in practice, ethical reflections on the use of GPS systems
Prof. Dr. Arne Manzeschke	Prof. of Ethics and Anthropology, Lutheran University of Applied Sciences Nürnberg	Expert was tasked with taking a more critical attitude towards the use of GPS systems. Introduction to the interplay of ethics, anthropology and technology, influence of technology on social life, human–machine interaction between users and GPS devices, advantages and disadvantages of GPS systems, GPS systems tend to support helpers more than patients

Ethics approval by the Göttingen University Medical Center ethics committee (AZ 29/4/22) was obtained.

### Qualitative interviews

To compare the group consensus of the CJ with individual preferences, qualitative interviews were conducted after the deliberative event concluded. This also allowed us to elicit the reasons individual participants had for their preferences and generated insights into the deliberative dynamic. Seventeen participants were asked to voluntarily participate in the interview study during their last session of the CJ. Participants received an e-mail detailing available time slots for the interviews, declarations of consent, and data protection provisions. 13 participants took part in an interview from 06/13/2022 to 08/10/2023 after providing consent. The gender ratio was balanced with 7 female and 6 male participants. Participant age ranged from 18 to 67 years. Interviews lasted between 17 and 45 min with an average of 28 min. 9 interviews were conducted via Zoom, 4 by telephone. One participant had technical issues, resulting in partial data loss.

We chose a semi-structured interview approach [2] to create a narrative-generating atmosphere. An interview guide with 20 questions was designed specifically for this study (see Supplementary File 1), which allowed the interviewer to switch between the questions and adapt them to the content of the conversation. These 20 questions covered five topic areas:

1. Attitudes towards GPS tracking devices for PwD.
2. The course of opinion development.
3. Factors influencing the formation of opinions.
4. The perception of the joint discussion.
5. Experiencing the CJ in an online setting and suggestions for improvement.

Participants were not systematically asked about prior personal or professional experience with dementia. The interview guide for the post-CJ interviews included open questions inviting reflection on participants’ views prior to the CJ and on whether personal experiences were brought into the deliberation and perceived as influential. As these questions were not designed to elicit a comprehensive account of prior exposure to dementia and interviews were conducted with a subset of jurors, variation in prior experience cannot be systematically described.

The interviews were transcribed via the software f4 [12], following transcription standards by [13]. To protect participant privacy, the transcripts were pseudonymized. The transcripts were coded and checked for intercoder reliability [9, 16]. For this purpose, the applicability and assignment of the individual categories were randomly tested with another doctoral student on five randomly selected transcripts. The findings from this process were also transferred to the coding guide to increase comprehensibility, and the units of analysis were adapted accordingly. The qualitative analysis of the interviews used [16] qualitative content analysis and was supported by the software ATLAS.ti [20, 23].

### Non-participant observation

After being announced to the participants and introducing herself, the project’s MD student joined the CJ as a silent observer, thus borrowing from the *non-participant observation* method of field note collection that de-emphasizes direct researcher involvement [15]. She joined all discussions with the exception of small group discussion exercises which were explicitly designed to give participants space to be among themselves without the presence of observers. Field notes were produced in the form of protocols, focusing on substantive attitudes of participants, the process of preference formation, consensus building among participants, and disagreement. The combination of deliberative methods, qualitative interviews, and non-participant observation allowed for a richer categorization of the attitudes of individual participants by giving insight not only into the substantive attitudes with respect to GPS tracking, but also into how participants with these attitudes interacted with others in deliberation.

## Findings

### Process

The online panels proceeded smoothly with minimal technical disruption. One participant reported occasional difficulties with handling the technical aspect of communicating via video chat but stated that this did not impact their ability to participate. Participants asked for an additional moderated session to further work on their policy recommendations document.

### Participant characteristics

Seventeen individuals participated in the CJ. Our selection criteria resulted in a balanced gender ratio of 9 women and 8 men, a near-even representation of four pre-defined age ranges, and a balanced ratio of university graduates and non-graduates (Table 2). However, 6 participants were still in education and might attain a university degree in the future. As is common for events involving policy discussions [24], participants skewed towards higher educational attainment, with 14 (82%) having attained the German general higher education entrance qualification or the technical college entrance qualification (*allgemeine Hochschulreife, Fachhochschulreife*), compared to the national average of 35% (Statistisches Bundesamt et al., [25]). 6 (35%) participants were students, 8 (47%) salaried employees, 1 (6%) self-employed, 1 (6%) retired, and 1(6%) unemployed. A large number of participants had a vocational connection to medicine and care, with 7 (41%) reporting to either work in medical or care professions or being in the process of obtaining a professional qualification for these fields. 3 (18%) participants reported a migration background (defined as at least one parent being born in a country other than Germany). 4 (24%) reported providing care for a dependent person. Prior exposure to the topic of the CJ was low, with 3 (18%) reporting experience with GPS tracking systems generally and 1 (6%) with tracking systems for PWD.

**Table 2:** Characteristics of participants and interviewees

	***CJ participants (n* = *17)***	***Interviewees (n* = *13)***
Age group (years) *Range: 18–67, median: 37*		
18–25	5 (29%)	4 (≈ 31%)
26–40	5 (29%)	1 (≈ 8%)
41–60	3 (18%)	4 (≈ 31%)
Above 60	4 (24%)	4 (≈ 31%)
Sex		
Female	9 (53%)	7 (≈ 54%)
Highest educational attainment		
Lower secondary school leaving certificate	3 (18%)	3 (≈ 23%)
Higher education entrance qualification	5 (29%)	2 (≈ 15%)
University degree	9 (53%)	8 (≈ 62%)
Occupation		
Salaried employee	8 (47%)	7 (≈ 54%)
Student	6 (29%)	4 (≈ 31%)
Self-employed	1 (6%)	1 (≈ 8%)
Unemployed	1 (6%)	
Retired	1 (6%)	1 (≈ 8%)
Other		
Vocational connection to medicine or care work	7 (41%)	*No data*
Migration background*	3 (18%)	*No data*
Has private care obligations	4 (24%)	*No data*
Has experience with GPS tracking systems	3 (18%)	*No data*
Has experience with GPS tracking systems for PWD	1 (6%)	*No data*

^*^At least one parent born without German citizenship

### Normative attitudes towards GPS tracking systems

The policy recommendations show a general tendency in favour of GPS tracking systems under the condition that the use is completely voluntary. Participants warned against any direct or indirect coercion to use trackers, for example when a care facility makes the consent to being tracked a requisite to being accepted as a patient. The report identifies four main areas of concern: a) ethical concerns, b) prevention and alternative approaches, c) data protection, data security, and privacy, and d) practical considerations related to the use of the technology. The ordering and categorization of the various ethical and technical dimensions of this issue follows the structure chosen by the participants for the policy recommendation document.

The qualitative analysis of the 13 interviews conducted after the CJ also showed a general tendency in favour of GPS use, but the more comprehensive exploration of participants’ reasons for their positions gave more depth to the minority position of those more skeptical of the technology.

In the following, we present an analysis of the consensus view produced by the CJ, explore how it came about with the help of a process analysis supported by the field notes, and contrast this view with the individual preferences that emerged from the qualitative interviews.

#### Differing conceptions of autonomy

The first area of concern tackled in the participant-produced policy recommendations was labelled’ethical concerns’ and focuses on the self-determination of PwD and the degree to which the use of tracking systems may increase or limit their autonomy, both for residential and home care. The report highlighted the potential conflict between maximising a PwD’s autonomy and the desire to utilise means of technological control to ensure their physical safety. With respect to this trade-off, the report described a split between two groups of participants. The first and larger group saw the tracking systems primarily as a tool enabling PwD to move around safely on their own, hence increasing their autonomy. The second, smaller, group disagreed with this framing and understood autonomy primarily as the uninhibited possibility to do as one pleases without outside interference or control, seeing freedom of movement as an inalienable right that should not be restricted due to illness. While the disagreement about framing tracking systems as tools of empowerment could not be resolved, agreement could be reached on the principle that a PwD’s freedom of movement should be maximised to the largest extent possible, but may be limited in cases in which PwD present a danger to themselves or others.

The report strongly emphasised the importance of consent. Participants differentiated between PwD fully capable of consent and those with a limited capacity. The report recommended that tracking systems should always be used in accordance with the PwD’s wishes and that PwD should be included in the decision-making process as early as possible. In particular, participants recommended the use of advance directives to specify a PwD’s wishes in advance (see the following section). The report approved the use of tracking systems, provided that their technical reliability is guaranteed, data security is ensured in accordance with German and European regulations and sufficient information and advice is available. Only in cases in which the PwD is no longer able to consent and presents a danger to themselves and others, the use of tracking systems may be authorised via court order.

The conflict between the benefits and risks of GPS tracking systems for PwD and the associated concepts of autonomy and surveillance to ensure the safety of PwD were addressed early on in the discussion in the CJ and emerged just after the opening presentation, where the conflicting issues of safety, surveillance and freedom were addressed for the first time in the summary of the presentation. The differing conceptions of autonomy in relation to PwD were also reflected in the use of different concepts of freedom, self-determination, and self-efficacy. The minority view of freedom of movement as an inalienable right did not emerge until the beginning of the preparation of the final document. One participant in particular argued strongly in its favour, and the analysis of the interviews showed that other participants also held this view. Initially, the discussions in the CJ also concentrated heavily on relieving the burden on caregivers. Halfway through the CJ, voices that emphasised a focus on PwD became more influential. This shift towards PwD was clearly reflected in the policy recommendations, which primarily focus on the PwD perspective as well.

The views expressed by participants in the interviews illuminate the split between the participants further, especially with respect to the differing conceptions of autonomy and how it is to be traded off against PwD’s physical safety. Some participants expressed a narrow understanding of autonomy. In particular with respect to freedom of movement, autonomy was regarded as the possibility to move freely and safely within a predefined controlled framework through GPS tracking systems, and caretakers would be called upon to restrict movement according to the risks for the PwD’s physical safety: *“That someone is freely determined and can move freely, i.e. within the framework of what is possible.” (Ms Giffard)*. For participants who held this view, ensuring the physical well-being of the PwD was the most important aspect: “*[…] it has often happened that people have disappeared, and I think it would be good if people could be found more quickly to prevent hypothermia, a fall into a river or something like that […]” (Ms Stelljes).* This group followed the majority view expressed in the policy recommendations and saw tracking systems as an option to increase the autonomy of PwDs, because tracking systems extend the range of localities and situations in which the physical safety of PwD is within acceptable bounds due to them being easily located and rescued should an emergency arise: *“[…]Now I'm tracking this person today because I want to know where they are today and then possibly intervene […] they can walk around for longer today because the weather is fine[…].” (Mr Vogt); “[…] I repeatedly see that people are being searched for. Firefighters, technical relief workers, and police are all deployed en masse to look for someone who has lost their way. And this [GPS tracking systems] is the most sensible way to avoid that” (Ms Lübke).*

A smaller group of interviewees followed the minority view mentioned in the policy recommendations and put forward a different understanding of autonomy that regards autonomy primarily as the unrestricted freedom of choice. They emphasized that autonomy should not take second place to safety, but rather be regarded as an inalienable right. Some emphasised this aspect by saying that a person with dementia, like any other person, has the right to expose themselves to a certain degree to danger: *“[…] One of them had raised this point […] if it is unsafe, i.e. if someone is on the road in city traffic and puts themselves in danger, then you can accept that […]” (Mr Wagner)*. One participant came to the conclusion that a restriction of these rights in home care, as in professional care, should only take place on the basis of a judicial decision*.* Other interviewees who agreed with this more extensive conception of autonomy were more willing to compromise should the physical safety of the PwD be at stake: *“I would still be cautious, but I've come to the conclusion that if it's really about safety, then perhaps GPS tracking should still be used […] provided the person has legal capacity and agrees to it.” (Ms Huber).* Adherents of the minority position tended to put the use of GPS tracking systems in the vicinity of a coercive measure while the policy recommendations emphasize the voluntary use of the technology: *“In the end, as we also found out […], […] you mainly move between self-determination and coercion” (Ms Huber).*

#### Advance care planning

The joint report highlighted the need for early preparation to enable PwD and their caretakers to take decisions that will enable PwD to live a self-determined life for as long as possible. These discussions extended beyond GPS tracking as a standalone technology and participants consistently situated GPS systems within broader questions of care planning, care coordination, and dementia awareness. In particular, the report focused on public awareness, education of affected persons and their caretakers, and the facilitation of advance care planning. In terms of providing education and information for those affected by dementia and their caretakers, participants expressed the view that access to a health professional could support PwD and their caretakers by coordinating care and providing information on dementia and assistive tools such as GPS tracking systems. The inclusion of the PwD in the decision-making process was a central aspect for the participants. The report stressed that the facilitation of advance care planning is a crucial component of enabling PwD to live a self-determined life. Participants suggested that, while the PwD still possesses the capacity to consent, a written care agreement involving PwD, caretakers, and health professionals should be established. This agreement was discussed as complementing advance care directives and as a way of addressing preferences regarding care and support as well as the use of assistive technologies, including GPS tracking. The report underlined the value of early planning to ensure that actions are taken in accordance with the PwD’s wishes and to ease the burden for the caretakers associated with having to make decisions on behalf of a PwD. It also highlighted the challenge of addressing situations in which preferences stated in an advance directive conflict with current expressions made by the PwD in a state of diminished mental capacity. Rather than offering a definitive solution, the report framed this issue as an open question to be addressed by wider public debate.

Views on care planning developed over the course of the CJ. Initially, the importance of care planning was briefly mentioned in the second expert presentation on opportunities and benefits of GPS tracking devices. Participants picked up this thread in group discussions and developed it into the idea of a broad care agreement with a network of health professionals. In the discussions, the proposal was presented as a solution addressing the question of how to better respond to the individual needs and preferences of PwD. This gelled with a line of reasoning spearheaded by one participant that sought to address the issue of wandering via human attention and companionship rather than technological solutions. The latter view was met by other participants with agreement in principle but also drew opposition due to its idealistic nature and lack of practicality. It did not make it into the final document.

The views expressed by the participants in the individual interviews reflect these concerns, especially with reference to the creation of an advance directive to cover the preference for assistance systems such as GPS tracking systems in the event that consent is no longer given. An important aspect that runs through the interviews is the voluntary use and application: *“[…] if you have recorded this[…] especially in a patient's advance directive, that you have a positive attitude towards it. Then I think that's a good solution in any case. Both for the person concerned and for everyone around, society, the relatives. […].”(Ms Giffard).*

It should be emphasized that almost all participants focused on the needs of PwD: *“[The] needs of the person with dementia is the most important thing.” (Ms Giffard).* Some participants, however, also focused on the burden for the caregiver and the relief that may be provided by assistive technologies: *“I think it was a bit different for all the participants, with some [you perhaps had] the feeling that they […] wanted to make the situation easier for the relative.” (Ms Giffard).*

### Data protection and privacy

The report acknowledges that locating a PwD via means of GPS tracking systems constitutes a potential encroachment on the right to privacy of PwD. It identifies location data as especially sensitive information and calls for strong protection mechanisms. The report warns of criminal misuse of location data and misappropriation of sensitive data by state entities. It also stresses that issues could arise when PwD visit locations that are subject to social taboos or places of which their caretakers might otherwise disapprove. The report strongly recommends to only collect such data that is necessary for the intended use case of locating lost PwD and further calls for effective encryption and strong authentication methods. Live-tracking without data storage is suggested as an ideal solution, but the text acknowledges that this might not always be technically feasible.

The document reports disagreement among participants about the appropriate level of precision for tracking. Some favored a precision of 100 m, some advocated for 10 m, some for as much precision as possible. The report calls for a broad societal debate over the appropriate balancing of security and privacy in this regard. It demands legislation that explicitly requires all providers of tracking solutions to comply with the European General Data Protection Regulation and to store data exclusively on servers within the European Union.

The aspect of data protection was explicitly mentioned at the beginning of the CJ by a participant and was brought up throughout in the subsequent discussions. Over the course of the CJ, this issue developed into a major topic that participants agreed to take on as one of the main issues of their report which featured very concrete solutions.

Following extensive discussions about privacy violations through trackers that extended into the third session, some participants proposed that the issue could be addressed by choosing an appropriate tracking radius limiting the accuracy of the tracking feature. However, as positions about the appropriate radius differed, no concrete recommendation was formulated.

A connected point of contention touched on the possibility of PwDs visiting places they would not like their caretakers to know about, such as brothels. This sparked an intense discussion about the appropriateness of using the example of visiting brothels in the report, which some participants regarded as crass. Participants agreed to solve the argument by referring to “stigmatized locations” (tabuisierte Orte) in the report.

In the individual interviews, participants expressed similar concerns about data protection and the associated invasion of privacy. All interviewees came to form a position on the topic of data protection and privacy violations, although they differed in how much normative weight they assigned. Some interviewees subordinated data protection under the physical safety of PwD: *“So […] for me it would be important to argue […] that you can also prioritise certain aspects, for example if a person runs away and puts themselves in danger, then I would simply classify that as more important than data protection, for example. Those would be important aspects that I would communicate.” (Mr Jansen) “That irritated me a bit. […] In the end, I thought that it was really more important to find the person, and that data protection can also be organised a bit, that there are rules for it.” (Ms Lübke).*

Others, on the other hand, rated data protection as very important and saw it as a critical aspect of the issue, especially as PwD cannot protect themselves in this regard. The protection of privacy and the associated sensitive data was then cited as a way of safeguarding their autonomy: *“People always blame data protection for this or that […] but it's actually about protecting people and their data from any negative consequences and I think that's also something you have to work out a bit and can do if you can justify it reasonably at the point.” (Mr Wibeau).*

### Practical considerations

The report recommends that tracking devices should be adaptable to the individual circumstances of the PwD, especially with respect to differences in rural vs. urban living environments, how dementia individually manifests for the individual, the preferences and needs of the PwD and their caretakers, as well as legal and ethical requirements. The report recommends data transmission via mobile phone signals due to the greater network coverage compared to local WLAN if the GPS signal is lost. It was also recommended that previously visited places should be stored temporarily to bridge signal losses. The report emphasizes the utmost importance of the reliability of the device, this includes a long battery life and ease of maintenance. The text further discusses the possibility to equip tracking devices with additional functions, such as navigation, emergency call and emergency alert functions. The report expresses concerns that such functions may not be used to their full potential by people with severe dementia but warns of potential privacy issues with automated emergency alert functions, especially insofar they rely on additional health data such as pulse and body temperature data. A fall sensor in conjunction with emergency alert functions was seen as a feasible compromise. The report recommends the standardization of tracking devices, the needs of people with disabilities should be taken into account in this regard.

The field notes show that participants were surprised by the large number of different devices on the market. As a result, they saw the necessity to formulate uniform requirements that each device should guarantee. They placed particular emphasis on the individual customisability of the devices (e.g. a customisable location radius), manageability and durability. This found entry into the recommendation document.

In the individual interviews, technical issues and solutions were mainly mentioned when interviewees recounted learning experiences taking place over the course of the CJ:: *“Like how this GPS tracking system is attached to people—in scare quotes. I found that very interesting and important to know in general, including the technical side behind it. But also […] what the current study situation is like.“(Ms Stelljes, emphasis added).*

### GPS tracking in the context of nursing staff shortages

One theme present in the interviews that was notably absent from the policy recommendation document as a result of the shift towards focusing on the needs of PwD was the potential effect of GPS tracking devices on professional nursing staff. Three participants explicitly mentioned nursing staff shortages in connection with GPS systems, although they did not propose concrete mechanisms by which GPS tracking would address staffing constraints. Rather, participants discussed GPS tracking in general terms as a technology that might make aspects of care easier or more manageable, while also expressing concern about the limits of technological approaches and the importance of human care. One interviewee saw the use of GPS systems as a possible support for nursing staff and a way to make nursing easier. Two others were weary of the technology potentially displacing human attention and emphasised the importance of human companionship: *“Using technology is easy. Especially when it is there and works well. It does not replace this human care and should not try to solve the care problem that currently exists politically. It can only be an emergency [option].” (Ms Huber).*

*“Staff shortages in care, the fact that there are more and more old people or older people/senior citizens, and fewer people to look after them and that alternative methods have to be used, so we have to rethink this completely.” (Ms Moser).*

### Emergent participant clusters: proponents, hesitants, skeptics

Based on the interviews, we identified the normative principles underlying individual positions, allowing us to cluster participants into three groups through an *ex post* thematic analysis of the interview transcripts (Table 3). The six participants who strongly supported the use of GPS systems are referred to as proponents. Their position was primarily driven by safety concerns for the well-being of PwD. They saw GPS trackers as enhancing PwD autonomy by extending the limits within which PwD could safely move about. This group emerged relatively early on in the CJ, with evidence from the interviews suggesting that these views were partly pre-existing. Four proponents (all either with professional care experience or caretakers for PwD themselves) held this stance from the start and remained consistent. The other two (both with a background in pedagogy) were initially uncertain but open to potential benefits of the technology. Over the course of the CJ, all six participants of this group became more informed about privacy and data security risks as well as the ethical tension between security and autonomy. Nevertheless, they maintained their stance prioritizing the physical safety of PwD and the support GPS trackers offer caregivers.

**Table 3:** Participant groups (categorized *ex post* based on thematic analysis) and their views

*Groups*	*Proponents (6)*	*Hesistant (4)*	*Sceptics (3)*
Participants (pseudonyms)	Ms Moser Ms Stelljes Ms Lübke Mr Vogt Ms Lützen Mr Donat	Mr Jansen Mr Mazur Ms Giffard Ms Wigand	Ms Huber Mr Wagner Mr Wibeau
General attitude	In favor of the use of GPS systems, emphasizes the physical safety of PwD	In favor of the use of GPS systems, with reservations	Skeptical of the use of GPS systems except in certain circumstances, highlights privacy risks
Key quotations	*“I think it’s so important to find a person and I’ve always said that this is the most important thing.”(Ms Lübke)*	“*The […] personal freedom […] which I see as self-realisation. I consider that very important. […]”(Mr Mazur)* *“I thought that maybe I don’t necessarily want that [for] myself when I have dementia later on, […] that people have access to where I might be. And somehow, that was a thought I didn’t like that much. Although, of course, I could imagine the advantages […]" (Ms Wigand)*	*“I […] always see that there is always a high potential for misuse of location data. […] it’s sensitive data; there’s actually little more sensitive data than knowing where people are […].” (Mr Wibeau)* *“We have already seen that safety is a fundamental issue for people. And freedom or self-determination are often put in the background […].” (Ms Huber)*
On Autonomy	+ See the need to protect the autonomy of PwD - However, PwD cannot assess possible risks due to their illness and therefore have to be restricted in their autonomy within reason	+ The will of the PwD takes priority - If there is considerable danger to the PwD’s life and to other people, there must be a restriction of autonomy	+ Advocate for the unconditional preservation of the autonomy of PwD - No restrictions
On Safety	+ GPS systems contribute to the safety of PwD + Safety takes precedence over other concerns	+ GPS systems contribute to the safety of PwD - At the same time, they see the dangers to the privacy of PwD and their autonomy	+ Over the course of the CJ, moved towards the position that GPS systems contribute to the safety of PwD - The gain in safety should never lead to a restriction of the autonomy of PwD
On data protection	+ Data protection is an essential point when talking about GPS tracking - It is not as important as the safety of PwD	+ Data protection is an important point when talking about GPS tracking - Data protection can interfere with effective tracking	+ Strong emphasis on data protection + Connect data protection to autonomy

The group of four participants (three students from various fields of study and one medical ethicist) who recognized benefits in GPS tracking systems but remained concerned about risks are referred to as ‘hesitants’. They were cautious about the use of such systems for PwD both at the beginning and at the end of the CJ. Over time, they acknowledged advantages such as benefitting the well-being of PwD and providing relief to caregivers. Their main normative reasons in favour of GPS systems were: 1) increased safe freedom of movement for PwD, reflecting a similar view of autonomy as the ‘proponents’ group; and 2) improved quality of life for PwD. However, they remained clearly concerned about surveillance and potential data misuse.

The last group, consisting of three participants (a philosopher, a computer scientist, and one with a background in education and rescue work) who were very critical of the use of GPS systems, is referred to as ‘skeptics’. Their stance was based on the unconditional preservation of the autonomy of PwD, avoidance of coercion, and protection of privacy from intrusions by the systems. They rejected a conception of autonomy as something that could be enhanced through surveillance and instead viewed it as the unimpeded freedom to act without external control.Two participants stated that they already held this view at the beginning of the CJ. Over time, they became more informed about advanced stages of dementia and the possible consequences of unaccompanied wandering for the well-being of PwD. As a result, they shifted slightly towards the collective view that GPS systems might be justified when necessary to prevent harm, especially if the alternative would involve even greater restrictions on autonomy. Their minority view, particularly with respect to data protection and privacy protections, was explicitly included in the final position paper.

## Limitations

CJs typically involve small samples and do not aim to produce statistically representative samples of the general population. However, they are often designed to approximate the demographic composition of a defined community. The present study did not adopt a design aimed at achieving community representativeness. Recruitment was based on self-selection and stratification by selected demographic characteristics, which limits the extent to which the findings can be interpreted as reflecting community-wide views. Instead, the findings should be understood as illustrating a range of normative positions and deliberative dynamics in discussions of the use of GPS tracking for PwD. Due to the complexity of the tasks and the technical operating requirements, no PwD were directly involved in the CJ.

A proportion of participants reported a vocational proximity to medicine or care. Although participants took part in the CJ without a designated stakeholder or expert role, such backgrounds may have influenced the perspectives articulated during deliberation and should be taken into account when interpreting the findings.

To ensure transparency in recruitment, our recruitment materials advertised the topic of the CJ. While this may have increased the likelihood of recruiting participants with prior beliefs about the issue, the deliberative process provided structured opportunities for participants to encounter a range of perspectives and information and to engage with the perspectives of their peers. Nonetheless, the potential influence of topic-specific recruitment on participant composition remains a limitation and should be taken into account when interpreting the findings.

Our sample skewed towards higher educational attainment, which may have affected opinion formation. While there is some research suggesting that the demographic make-up of self-selected online panels closely resembles those of in-person meetings in terms of socioeconomic background [8], these findings will need to be systematically evaluated at a larger scale before robust conclusions can be drawn.

Facilitation was provided by members of the research team rather than by an independent third-party facilitator. This may have shaped how participants perceived the facilitator’s role and neutrality, and should therefore be taken into account when interpreting the deliberative process.

Our interview study did not include all participants of the deliberative event. Two participants did not respond to the invitation to join the interview study, and two declined to participate due to time constraints. This may have resulted in a missed opportunity to discover unvoiced dissent. It is important to note however, that the most outspoken members of the minority group (sceptics) did partake in the interviews.

## Discussion

Despite fundamental differences on the meaning of autonomy, participants were able to come to a robust consensus on most substantive issues, such as the importance of informed consent, the need to raise public awareness about dementia, the education of PwD and their caretakers, the facilitation of advanced care planning, strict data protection, requirements for the reliability of devices, and the option to customize devices to suit individual needs and preferences. Participants were also able to agree to disagree on other matters such as the most appropriate accuracy of the tracking devices and differing prioritizations in the trade-off between physical safety and the risk of privacy violations. For these contentious matters, participants were able to agree on a common description of their differences, showcasing the ability to find agreement on a meta-level. This process of successful consensus production deserves closer attention and will be covered in a separate contribution.

Remarkably, the issues raised by participants closely tracked the expert discourse on the matter without participants having received in-depth instruction on the state of the debate. This was especially apparent in the pivotal nature of differing conceptions of autonomy brought forward by different groups of participants. This mirrors the focus of the bioethical literature on this point which emphasises the dual effect of a technology that can both enhance PwD autonomy by enabling greater freedom of movement within acceptable bounds of safety, and may diminish it by inducing PwD to change where they move due to being monitored [11]. Beyond this, participants further confronted a topic largely ignored by the expert discourse by addressing practical concerns related to the technical capabilities of the devices such as their tracking accuracy (ibid.). This result underscores the utility of consulting lay people to advance bioethical debates [22].

The 17 participants of the CJ were able to create a nuanced recommendation for action over 5 online sessions via the video conferencing software Zoom. This recommendation contains differentiated, well-considered positions and illuminates complex ethical areas of conflict in the context of the use of GPS tracking systems for PwD suffering from a loss of orientation. This promising result adds to the growing body of literature showing that online deliberation is not necessarily inferior to face-to-face deliberation in terms of increasing participants’ knowledge and enabling fair and constructive discussions [10]. Our research further adds to the still-scarce literature on the feasibility and quality of deliberative meetings facilitated by videocommunication software [8, 28].

By combining the deliberative setting of the CJ with qualitative interviews, it was possible to gain a better understanding of the consensus-building process. In particular, the consideration of minority opinions and the inclusion of these in the consensus-building process can be better assessed. Furthermore, the individual interviews allowed a comparison of collective and individual preferences while the non-participant observation enabled us to track the formation of participant clusters with similar preferences and attitudes. With the help of the interviews, the initial division between the majority and minority group mentioned in the policy document can be more precisely categorized into three groups – proponents, hesitant, sceptics. The divergence of the groups’ perspectives and the convergence of differing opinions became apparent and could be traced to contrasting conceptions of PwD autonomy.

## Supplementary Information

Supplementary Material 1.

## Data Availability

The datasets used and/or analysed during the current study are available from the corresponding author on reasonable request.
