# Supplementary material for: Ethical perspectives on GPS tracking for people with dementia: insights from an online citizens’ jury
Source: BMC Med Ethics. 2026 Mar 3;27:74. doi: 10.1186/s12910-026-01423-5 (PMC13063461; doi:10.1186/s12910-026-01423-5)
Supplement: Supplementary file 1 — Supplementary Material 1. [file 12910_2026_1423_MOESM1_ESM.docx]

# **Supplementary File 1**

## **Semi-structured Interview Guide**

**Manuscript Title:** Ethical Perspectives on GPS Tracking for People with Dementia: Insights from an Online Citizen Jury

This semi-structured interview guide was developed specifically for the study reported in the above manuscript. It was designed as a follow-up instrument to the Bürgerforum GPS-Ortungssysteme für Menschen mit Demenz (Citizens’ Forum on GPS Tracking Systems for People with Dementia) in order to explore participants’ processes of opinion formation, their evaluation of the forum format, and their reflections on the ethical aspects of GPS tracking in dementia care. The interviews were conducted in German, and the original interview guide was written in German. The version provided here is an English translation prepared for publication. Written informed consent for interview participation was obtained from all participants prior to the interviews.

### Interview Introduction (to be read aloud to participants)

The aim of this interview is to examine how opinions were formed within the Citizens’ Forum. I would like to point out that participation in this interview is voluntary. Refusing to participate will not result in any disadvantage for you. The interview may be interrupted at any time. I will record the conversation and then transcribe it. The data will be stored on the servers of the University Medical Center Göttingen and will be accessible only to the research team. You may contact us at any time and ask us to delete the data. Do you agree that I may now start the recording?

### Interview Questions

1. As you already know, the Citizens’ Forum dealt with the decision on the use of GPS tracking systems for people with dementia. What was your opinion on this topic before the Citizens’ Forum?
2. What were your main reasons for holding this opinion before the Citizens’ Forum?
3. How did your opinion develop during the course of the Citizens’ Forum?
4. How did the Citizens’ Forum influence your opinion?
5. Was there a specific event that prompted you to reconsider or reinforce your opinion? (For example, something another participant said?)
6. During the Citizens’ Forum, were you able to gain new arguments to support or change your opinion? If so, what were these arguments?
7. Where did these new arguments come from? (From other participants, experts, or the group discussion?)
8. How did you perceive the group discussion?
9. How did you perceive the other participants?
10. How would you assess your role in the group discussion?
11. Were there particular ways in which other participants’ arguments stimulated your thinking?
12. To what extent do you believe that other participants influenced your opinion?
13. To what extent do you think you brought in your personal experiences during the discussion, or that personal experiences were contributed by other participants? Do you think these personal experiences influenced the course of the Citizens’ Forum?
14. Based on the experience you gained in the Citizens’ Forum, how would you personally decide regarding the use of GPS-supported tracking systems? (At this point, the reasons for such decisions should be asked about.)
15. In your view, which reasons are particularly important to convince outsiders (those not part of the Citizens’ Forum) of your opinion?
16. What makes these reasons the most important ones?
17. What is your view of the joint decision that was reached?
18. How did you perceive the format of the online event?
19. Which aspects could be improved?
20. What do you think of this format of joint decision-making within a Citizens’ Forum for political decisions?
